# Family influences on children's physical activity and fruit and vegetable consumption

**DOI:** 10.1186/1479-5868-6-34

**Published:** 2009-06-16

**Authors:** Natalie Pearson, Anna Timperio, Jo Salmon, David Crawford, Stuart JH Biddle

**Affiliations:** 1 School of Sport, Exercise and Health Sciences, Loughborough University, UK; 2 Centre for Physical Activity and Nutrition Research, School of Exercise and Nutrition Sciences, Deakin University, Australia

## Abstract

**Background:**

There is evidence of a clustering of healthy dietary patterns and physical activity among young people and also of unhealthy behaviours. The identification of influences on children's health behaviors, particularly clustered health behaviors, at the time at which they develop is imperative for the design of interventions. This study examines associations between parental modelling and support and children's physical activity (PA) and consumption of fruit and vegetables (FV), and combinations of these behaviours.

**Methods:**

In 2002/3 parents of 775 Australian children aged 10–12 years reported how frequently their child ate a variety of fruits and vegetables in the last week. Children wore accelerometers for eight days during waking hours. Parental modelling and parental support (financial and transport) were self-reported. Binary logistic and multinomial logistic regression analyses examined the likelihood of achieving ≥ 2 hours of PA per day (high PA) and of consuming ≥ 5 portions of FV per day (high FV) and combinations of these behaviors (e.g. high PA/low FV), according to parental modelling and support.

**Results:**

Items of parental modelling and support were differentially associated with child behaviours. For example, girls whose parents reported high PA modelling had higher odds of consuming ≥ 5 portions of FV/day (OR = 1.95, 95% CI = 1.32–2.87, p < 0.001). Boys whose parents reported high financial support for snacks/fast foods had higher odds of having 'high PA/low FV' (OR = 2.0, 95% CI = 1.1–3.7).

**Conclusion:**

Parental modelling of and support for physical activity and fruit and vegetable consumption were differentially associated with these behaviours in children across behavioural domains and with combinations of these behaviours. Promoting parents' own healthy eating and physical activity behaviours as well encouraging parental modelling and support of these behaviours in their children may be important strategies to test in future research.

## Background

The importance of examining influences on children's physical activity and fruit and vegetable consumption is underpinned by the recognition that lifestyles including adequate levels of physical activity and high intakes of fruit and vegetables have positive health benefits and important health-protective effects [1,2]. Despite this, there is evidence that many children have low levels of physical activity [3,4] and eat less fruit and vegetables than is recommended for health [5,6]. Evidence suggests that physical activity behaviours and fruit and vegetable intake are established in childhood [7-10] and may persist into adulthood [11,12]. Furthermore, there is evidence of a clustering of healthy dietary patterns and physical activity among children [13,14], and also of unhealthy behaviours such as low fruit and vegetable consumption and TV viewing [15]. The tendency for such health behaviours to aggregate has important implications for health promotion, highlighting the need for effective multiple behaviour interventions to improve diet and physical activity among young people [16].

Given such evidence, the identification of influences on children's health behaviours, particularly clustered health behaviours, at the time at which they develop is imperative for the design of interventions. Socialization of health-related behaviours occurs within the family, with parents' beliefs, attitudes and behaviours substantially affecting children's health behaviours [17]. Previous research suggests that adults who have a high level of concern for health are likely to make several consistent healthy lifestyle choices, which include regular exercise and reasonable food choice [18]. For example, active adults have been found to eat healthier diets, consume fewer servings of fried foods and sweets, and more servings of fruits and vegetables [19-21]. Such adults may also encourage their children to do the same. However, there is a lack of research examining family correlates of multiple health behaviours among children, particularly across behavioural domains (e.g. considering potential links between parental physical activity modelling and children's dietary behaviours, such as consumption of fruit and vegetables). Examining family correlates of children's behaviour across behavioural domains may provide an insight into whether parental behaviour transfers across to different health behaviours in children. Such information will provide greater clarity and depth of understanding of the dynamics of the relationship between parental and child behaviour.

Studies examining correlates of children's physical activity and fruit and vegetable intake tend to focus on associations within the same behavioural domains. For example, recent reviews have shown that parental support for physical activity was consistently positively associated with children's physical activity [22] and that indicators of parental support for fruit and vegetable consumption, such as encouragement and facilitation, were positively associated with children's fruit and vegetable intakes [23-25]. In addition, parental modelling of fruit and vegetable consumption has been shown to be positively associated with children's fruit and vegetable consumption [23] and parental physical activity modelling has been shown to be positively associated with children's physical activity, though the latter was evident only amongst boys [22]. Although there is a growing body of literature examining influences on children's physical activity [22,26] and children's fruit and vegetable consumption [23,27], studies examining influences on multiple health behaviours are lacking. Examining combinations of children's health behaviours (for example, those children with high physical activity levels and low fruit and vegetable consumption) and influences on these, is potentially useful as these behaviours do not occur in isolation in the real world and such data will provide a first step towards the development of effective primary prevention strategies [28].

To our knowledge there is currently no published research examining associations between parental modelling and support and children's combined physical activity and fruit and vegetable consumption (e.g. is parental physical activity modelling associated with 'high physical activity and high fruit and vegetable consumption'?). The aim of the present study, therefore, is to examine cross-sectional associations between a) parental modelling and support and children's physical activity and fruit and vegetable consumption individually, and b) between parental modelling and support and children's combined physical activity and fruit and vegetable consumption.

## Methods

Cross-sectional data was collected between November 2002 and December 2003 as part of the Health, Eating and Play Study: a study investigating family environmental influences on changes in children's eating, physical activity and obesity throughout childhood and adolescence. The parent responsible for the daily care of the child completed a questionnaire and the children each wore an accelerometer to provide an objective measure of physical activity. Ethical approval was received from Deakin University, the Department of Education and Training Victoria, and the Catholic Education Office.

### Participants

Children aged 10–12 years were recruited from 17 State or Catholic primary schools within the Greater Melbourne and Geelong areas in Victoria, Australia [29]. Schools included those with enrolments of greater than 200 students and were randomly selected from postcodes with high, medium and low socio-economic status based on census data. Recruitment procedures have been reported previously [29]. In total, 2085 children were provided with information about the study and with consent forms to take home to their parents. Under existing ethical guidelines, only families who provided active written consent were eligible to participate.

### Measures

#### Sociodemographics

Parents provided demographic information including their age, sex, language usually spoken at home, marital status, education level and employment status. In addition, parents provided the date of birth and sex of their child enrolled in the study.

#### Family environment

Parents were asked seven questions about the frequency with which they modelled physical activity or healthy eating, or provided transport or financial support to their child for physical activity and dietary behaviours. For example, 'how often did you and/or the co-carer do physical activity, sport or exercise together with the child in the past week?' Question wording and response scales are presented in Additional file 1, Table S1. Due to low numbers in a number of the response categories, parents' responses were collapsed into either two or three categories as described in Additional file 1, Table S1.

#### Physical activity

Children's physical activity was assessed using accelerometers (Actigraph Model AM7164-2.2C, Manufacturing Technology Inc [MTI], Florida, USA). Except for during water-based activities and while sleeping, children were asked to wear the accelerometer on their right hip for eight days. Average duration of moderate- and vigorous-intensity physical activity per day was computed only for children who wore the accelerometer for a minimum of four days including a weekend day (the threshold for estimating children's habitual physical activity using accelerometers, [30]), defined as days on which accelerometer counts were between 10, 000 and 20 million [31].

To estimate the time spent per day in moderate intensity physical activity (3.0–5.9 metabolic equivalent of rest [METs]) and vigorous intensity physical activity (6.0+ METs), age-specific movement count thresholds [32] were applied to the accelerometer data. Time per day in moderate-to-vigorous physical activity (MVPA) was derived by summing and averaging these values across valid days. The proportion of children meeting physical activity recommendations for young people [33] was calculated according to whether they performed an average of ≥60 mins/day of MVPA. As many children in the sample (~90%) exceeded the 60 min/day recommendation and the Australian guidelines recommend that children participate in 'up to several hours' of MVPA each day [34], time spent in MVPA per day was dichotomized into <2 h/day (low) or ≥2 h/day (high).

#### Fruit and vegetable intake

Parents were asked how often their child ate 14 different fruits (e.g. apples, bananas, grapes) or types of fruit (e.g. stone fruit, citrus fruit, dried fruit) and 13 different vegetables (e.g. peas or beans, cauliflower, squash) or types of vegetables (e.g. mixed vegetables, root vegetables) in the last week, excluding potatoes. These items were adapted from the most recent Australian National Nutrition Survey (NNS) [35] and showed good to excellent reliability (ICC = 0.44–0.96) when tested over 2–3 weeks among a subset of 93 parents. An eight-point response scale was provided for each fruit and vegetable item; responses were recoded (scores presented in parentheses) and summed to compute total frequency of fruit and vegetable consumption/day, respectively: 4 or more times/day (4); 3 times/day (3); twice/day (2); once/day (1); 4–6 times/week (0.714); 2–3 times/week (0.357); once/week (0.143); not eaten (0), as previously reported [36]. For the purpose of this study each item was summed to calculate the frequency of consumption of 'fruits and vegetables' (FV) per day. Guided by the current guidelines for fruit (≥2 portions/day) and vegetable (3–5 portions/day) consumption for Australian children in these age groups [37], the total daily frequency of FV intake was dichotomized into <5 times/day (low) or ≥5 times/day (high).

### Statistical Analyses

Initial analyses were undertaken using SPSS statistical software version 14.0. Descriptive statistics were used to describe parent characteristics and the distribution of predictor and outcome variables. The proportion of children achieving ≥2 hours/day of MVPA and the proportion consuming fruit and vegetables ≥5 times/day were compared by gender and maternal education using Pearson Chi-square (χ^2^) tests of significance. As there were significant differences by gender and by maternal education, all further analyses were conducted separately for boys and girls, and adjusted for maternal education.

Bivariate binary logistic regression analyses were conducted to examine the odds of achieving ≥2 hours/day of MVPA (high), and of consuming fruit and vegetables ≥5 times/day (high), respectively, with measures of parental modelling and support entered as independent variables. Four categories were created combining physical activity and fruit and vegetable consumption: high PA/high FV; high PA/low FV; low PA/high FV; low PA/low FV. Multinomial logistic regression analyses were conducted to examine the likelihood of being in each of these categories according to parent modelling and support variables. The 'low PA/low FV' category was used as the referent category. All regression analyses were undertaken using Stata 8 (Stata Corp, College Station TX, 2003) to allow for potential clustering by schools. Parental modelling and parental support items that were significantly associated with combinations of physical activity and fruit and vegetable consumption in the multinomial logistic regression analyses were entered into multivariate multinomial logistic regression models.

## Results

### Participant profile

Active consent was received for 947 children (46% response rate). Due to incomplete physical activity and FFQ data, 172 children were excluded from analyses. The analyses presented here are based on 775 children: 354 boys (mean age 11.3, SD 0.6, years) and 421 girls (mean age 11.2, SD 0.6, years). Responding parents were most commonly female (84%), reported speaking English at home (87%) and were married or co-habiting (86%). In addition, 24% of mothers had not completed secondary school, 30% were secondary school educated, 10% had a technical/trade school certificate/diploma and 36% were university/tertiary educated.

### Children's physical activity and fruit and vegetable intake

Table 1 shows the distribution of outcome variables for boys and girls. Compared to girls, greater proportions of boys had high levels of MVPA and were classified within the 'high PA/low FV' group. In contrast, greater proportions of girls had a high frequency of fruit and vegetable intake and were classified within the 'low PA/high FV' group and the 'low PA/low FV group', compared to boys.

**Table 1:** Distribution of outcome variables.

***Outcome variables***	***Total*** ***%***	***Gender (%)***		
			
***n = 775***		**Boys** **(n = 354)**	**Girls** **(n = 421)**	
**Physical activity**				***
≤ 2 hours MVPA/day (low)	49.4	37.3	59.6	
≥ 2 hours MVPA/day (high)	50.6	62.7	40.4	
**Fruit and vegetable consumption**				**
≤ 5 portions/day (low)	47.6	53.1	43.0	
≥ 5 portions/day (high)	52.4	46.9	57.0	
**Groups**				
High PA/High FV	25.8	28.2	23.8	
High PA/Low FV	24.8	34.5	16.6	***
Low PA/High FV	26.6	18.6	33.3	***
Low PA/Low FV	22.8	18.6	26.4	***

* p < 0.05; ** p < 0.01; ***p < 0.001: Pearson's chi-square analyses between boys and girls.

### Parent modelling and support

Additional file 1, Table S1 shows the distribution of parental modelling and support variables by child gender. Greater proportions of boys received high parental support for physical activity compared to girls. However, there were no differences in parental modelling or support of eating behaviours by child's gender.

### Associations between parental modelling and support, and physical activity and frequency of fruit and vegetable consumption

Logistic regression analyses showed that several of the parental modelling and support items were significantly associated with children's physical activity and fruit and vegetable consumption (Additional file 2, Table S2). Compared to girls whose parents reported low physical activity modelling, girls whose parents reported high physical activity modelling had higher odds of consuming fruit and vegetables ≥5 times/day. Boys and girls whose parents reported high breakfast modelling had higher odds of consuming fruit and vegetables ≥5 times/day, compared to those with parents who reported low breakfast modelling.

Compared to girls whose parents reported low financial support for physical activity, girls whose parents reported high financial support had higher odds of having high levels of MVPA. Among boys, those whose parents reported high support (transport) for fast food had lower odds of having high levels of MVPA compared to those whose parents reported low support.

Several parental modelling and support items were associated with combinations of MVPA and fruit and vegetable consumption in the multivariate multinomial logistic regression analyses shown in Additional file 3, Table S3 and Additional file 4, Table S4. High physical activity modelling was positively associated with being in the high PA/high FV category among both boys and girls. Transport related support for physical activity was also positively associated with being in the high PA/high FV category among girls. In addition, financial support for snacks and fast foods among boys, and high parental modelling during dinner among girls, were positively associated with being in the high PA/low FV category. None of the variables examined were associated with the low PA/high FV category.

## Discussion

This study examined associations between parental modelling and support and children's physical activity, frequency of consumption of fruit and vegetables and combinations of these behaviours. Current research tends to focus on examining associations between parent and child behaviours in the same behavioural domain (e.g. parental physical activity modelling and children's physical activity), and on single health behaviours. Findings of the present study justify further research examining associations between parental and child behaviours across different domains, and between parental behaviour and clustered health behaviours of children. Such research will provide a better understanding of the transferability of behavioural correlates to different health behaviours, as well as provide a greater depth of understanding about the dynamics of the relationship between parent and child behaviour. Our findings give weight to the importance of addressing parental behaviours in interventions aimed at promoting healthy behaviours among children. They revealed several significant associations that were not always in the expected direction and in most cases differed for boys and girls, suggesting that intervention strategies may need to be tailored according to the child's gender.

Parental modelling appears to be a consistent correlate of positive health behaviours for boys and girls. Results of the present study suggest that parents who engage in healthy behaviours with their children are more likely to have children who also exhibit positive health behaviours and not necessarily in the same behavioural domain. For example, we found that parental physical activity modelling was positively associated with high FV consumption among girls and with 'High PA/High FV' among boys and girls in this study. In addition, parental modelling during breakfast was positively associated with high FV consumption among boys and girls. This finding supports previous research of positive associations between parental modelling and children's fruit and vegetable consumption [23]. Eating meals and being physically active together are important opportunities for parents to be positive role models to their children. Although not assessed in the current study, parents should also be encouraged to target their own dietary and physical activity behaviours as indirect modelling has also been found to correlate with those respective behaviours among children [13,23].

Parental support for physical activity appears to be an important correlate of girls' health behaviours. Financial support for physical activity was positively associated with girl's MVPA and transport-related support for physical activity was positively associated with girl's 'High PA/High FV'. Such findings support previous research showing that parents who support and facilitate their child's physical activity are more likely to have children who are active [26]. Among boys, parental support in taking their child to a fast food restaurant was inversely associated with MVPA and with 'High PA/High FV' (before adjustment). In contrast, parents who provided money to their children to buy snacks and treats were more likely to have boys who had 'High PA/Low FV'. It may be that parents are providing money for snacks and treats after their child has been active (e.g. money for vending machines after a training session at a sports centre). One strategy for parents would be to make fruit and vegetable snacks available and ready for children to eat after activities. Recent reviews show that availability of FV and parental facilitation are positively associated with children's consumption of fruits and vegetables [23,27].

Strengths of the study include the sample of children from a range of socioeconomic and family environment backgrounds, the objective assessment of physical activity, and the use of health guidelines to determine combinations of groups. This study is limited by its cross-sectional design and the generalisability of the results is limited because our sample was drawn from urban schools in Victoria, Australia and so does not represent the population at large. In addition, research has shown weak to moderate associations between parent and child reports of fruit and vegetable intake [38]. However, parent report of children's frequency of consumption of fruit and vegetables was used in this study because previous research has shown that primary-school aged children may have limited recall skills and ability to estimate and indicate portion sizes and lack of knowledge of foods [39] to provide valid information about food frequency, resulting in overestimation [38,40]. The current study focused only on social influences from parents, and individual factors and the broader physical environment are likely to provide additional dimensions of influence which contribute to or explain these specific behaviours in children. Longitudinal studies that identify whether combinations of behaviours track over time and whether parental influences are temporally associated with change in these behaviours are required.

## Conclusion

This study found that parental modelling of and support for physical activity and fruit and vegetable consumption were differentially associated with these behaviours in children across behavioural domains and with combinations of these behaviours. Promoting parents' own healthy eating and physical activity behaviours as well encouraging parental modelling and support of these behaviours in their children may be important strategies to test in future research.

## Competing interests

The authors declare that they have no competing interests.

## Authors' contributions

NP analyzed the data and conceived and drafted the original manuscript. DC designed the overall Health Eating and Play study (HEAPs) project, and provided critical feedback on drafts. AT, JS and SJHB provided critical feedback on drafts. All authors read and approved the final manuscript.

## Supplementary Material

Additional file 1 **Table S1. Description and distribution of parental modelling and support items**. The data provided describe the distribution of the modelling and support items used in the study. Click here for file

Additional file 2 **Table S2. Parental modelling and support items and the odds of achieving ≥ 2 hours/day MVPA and ≥ 5 fruit and vegetables/day among boys and girls**. The data provided present results from the binary logistic regression analysis examining the odds of children achieving ≥ 2 hours/day MVPA and ≥ 5 fruit and vegetables/day according to parental modelling and support items. Click here for file

Additional file 3 **Table S3. Parental modelling and support items and the likelihood of combinations of physical activity and fruit and vegetable consumption among boys**. The data provided present results from the multivariate multinomial logistic regression analyses examining the likelihood of combinations of physical activity and fruit and vegetable consumption among boys according to parental modelling and support items. Click here for file

Additional file 4 **Table S4. Parental modelling and support items and the likelihood of combinations of physical activity and fruit and vegetable consumption among girls**. The data provided present results from the multivariate multinomial logistic regression analyses examining the likelihood of combinations of physical activity and fruit and vegetable consumption among girls according to parental modelling and support items. Click here for file
