# Supplementary material for: Family influences on children's physical activity and fruit and vegetable consumption
Source: Int J Behav Nutr Phys Act. 2009 Jun 16;6:34. doi: 10.1186/1479-5868-6-34 (PMC2703614; doi:10.1186/1479-5868-6-34)
Supplement: Additional file 1 — Table S1. Description and distribution of parental modelling and support items. The data provided describe the distribution of the modelling and support items used in the study. [file 1479-5868-6-34-S1.doc]

**Table S1.** Description and distribution of parental modelling and support items.

| **Parental modelling and support variables** |  | **Boys %**  ***(n=354)*** | **Girls %**  ***(n=421)*** |
| --- | --- | --- | --- |
|  |  |  |  |
| ***Parental modelling of physical activity*^1^** |  |  |  |
| How often did you and/or the co-carer do physical activity, sport or exercise together with the child? | Low | 74.9 | 75.2 |
|  | High | 25.1 | 24.8 |
|  |  |  |  |
| ***Parental modelling of eating behaviours*** |  |  |  |
| How often did you and/or the co-carer eat breakfast at home with the child? **^1^** | Low | 27.7 | 27.8 |
|  | High | 72.3 | 72.2 |
|  |  |  |  |
| How often did you and/or the co-carer eat dinner at home with the child? **^2^** | Low | 29.4 | 25.9 |
|  | High | 70.6 | 74.1 |
|  |  |  |  |
| ***Parental support for physical activity (transport and financial)* ^1^** |  |  |  |
| How often did you and/or the co-carer take the child to sports/training/lessons? (transport) | Low | 48.6 | 62.0 *** |
|  | High | 51.4 | 38.0 *** |
|  |  |  |  |
| How often did you and/or the co-carer provide money for the child’s sport or physical activity? (financial) | Low | 50.9 | 61.8 ** |
|  | High | 49.1 | 38.2 ** |
|  |  |  |  |
| ***Parental support for eating behaviours (transport and financial)* ³** |  |  |  |
| How often did you and/or the co-carer provide money for the child’s snacks/treats/fast foods? (financial) | Low | 32.3 | 32.8 |
|  | Medium | 25.1 | 26.6 |
|  | High | 42.6 | 40.6 |
|  |  |  |  |
| How often did you and/or the co-carer take the child to fast food restaurants? (transport) | Low | 44.1 | 40.4 |
|  | Medium | 37.5 | 39.5 |
|  | High | 18.4 | 20.1 |

^1^ Low = ‘never or rarely’ and ‘once a week’; High = ‘2-3 times a week’ to ‘every day’. Six response categories were (1) ‘never/rarely’, (2) ‘less than once/week’, (3) ‘once a week’, (4) about 2-3 times/week’, (5) ‘about 4-6 times/week’, (6) ‘every day’

^2^ Low = ‘never or rarely’ to ‘4-6 times a week’; High = ‘every day’ Six response categories were (1) ‘never/rarely’, (2) ‘less than once/week’, (3) ‘once a week’, (4) about 2-3 times/week’, (5) ‘about 4-6 times/week’, (6) ‘every day’

³ Low = ‘never or rarely’; medium = ‘less than once a week’; High = ‘once a week’ to ‘every day’ Six response categories were (1) ‘never/rarely’, (2) ‘less than once/week’, (3) ‘once a week’, (4) about 2-3 times/week’, (5) ‘about 4-6 times/week’, (6) ‘every day’

*p<0.05; ** p<0.01; ***p<0.001: Pearson’s chi-square analyses between boys and girls.
