# Supplementary material for: Family influences on children's physical activity and fruit and vegetable consumption
Source: Int J Behav Nutr Phys Act. 2009 Jun 16;6:34. doi: 10.1186/1479-5868-6-34 (PMC2703614; doi:10.1186/1479-5868-6-34)
Supplement: Additional file 2 — Table S2. Parental modelling and support items and the odds of achieving ≥ 2 hours/day MVPA and ≥ 5 fruit and vegetables/day among boys and girls. The data provided present results from the binary logistic regression analysis examining the odds of children achieving ≥ 2 hours/day MVPA and ≥ 5 fruit and vegetables/day according to parental modelling and support items. [file 1479-5868-6-34-S2.doc]

**Table S2**. Parental modelling and support items and the odds of achieving > 2hours/day MVPA and > 5 fruit and vegetables/day among boys and girls.

|  | **Boys (n=354)** | | | | | | **Girls (n=421)** | | | | | |
| --- | --- | --- | --- | --- | --- | --- | --- | --- | --- | --- | --- | --- |
|  | **MVPA (> 2 hours/day)** | | | **Fruit and Vegetables (>5/day)** | | | **MVPA (> 2 hours/day)** | | | **Fruit and Vegetables (>5/day)** | | |
|  | **OR** | **95% CI** |  | **OR** | **95% CI** |  | **OR** | **95% CI** |  | **OR** | **95% CI** |  |
| I/we did physical activity, sport or exercise together with the child |  |  |  |  |  |  |  |  |  |  |  |  |
| *Low*ª | 1 |  |  | 1 |  |  | 1 |  |  | 1 |  |  |
| *High* | 1.46 | 0.96-2.23 |  | 1.37 | 0.89-2.10 |  | 1.36 | 0.90-2.07 |  | 1.95 | 1.32-2.87 | *** |
| I/ we ate breakfast at home together with the child |  |  |  |  |  |  |  |  |  |  |  |  |
| *Low*ª | 1 |  |  | 1 |  |  | 1 |  |  | 1 |  |  |
| *High* | 0.85 | 0.53-1.35 |  | 1.53 | 0.99-2.36 | * | 0.86 | 0.57-1.31 |  | 1.66 | 1.02-2.69 | * |
| I/ we ate dinner at home together with the child |  |  |  |  |  |  |  |  |  |  |  |  |
| *Low*ª | 1 |  |  | 1 |  |  | 1 |  |  | 1 |  |  |
| *High* | 0.92 | 0.60-1.41 |  | 1.18 | 0.76-1.84 |  | 1.33 | 0.82-2.16 |  | 1.27 | 0.85-1.89 |  |
| I/we took the child to sports training/lessons |  |  |  |  |  |  |  |  |  |  |  |  |
| *Low*ª | 1 |  |  | 1 |  |  | 1 |  |  | 1 |  |  |
| *High* | 1.17 | 0.73-1.87 |  | 1.12 | 0.65-1.93 |  | 1.42 | 0.98-2.07 |  | 1.20 | 0.88-1.64 |  |
| I/we provided money for sport or physical activity that the child did |  |  |  |  |  |  |  |  |  |  |  |  |
| *Low*ª | 1 |  |  | 1 |  |  | 1 |  |  | 1 |  |  |
| *High* | 1.34 | 0.75-2.37 |  | 1.27 | 0.73-2.20 |  | 1.56 | 1.08-2.26 | * | 0.88 | 0.64-1.20 |  |
| I/we provided money to the child to buy snacks, treats or fast food |  |  |  |  |  |  |  |  |  |  |  |  |
| *Low*ª | 1 |  |  | 1 |  |  | 1 |  |  | 1 |  |  |
| *Medium* | 1.60 | 0.98-2.61 |  | 0.79 | 0.41-1.53 |  | 1.36 | 0.82-2.27 |  | 1.08 | 0.58-2.02 |  |
| *High* | 1.61 | 0.97-2.70 |  | 0.69 | 0.41-1.15 |  | 0.68 | 0.42-1.11 |  | 0.88 | 0.46-1.69 |  |
| I/we took the child to fast food restaurants |  |  |  |  |  |  |  |  |  |  |  |  |
| *Low*ª | 1 |  |  | 1 |  |  | 1 |  |  | 1 |  |  |
| *Medium* | 0.69 | 0.42-1.13 |  | 0.77 | 0.49-1.20 |  | 0.84 | 0.50-1.43 |  | 0.77 | 0.46-1.29 |  |
| *High* | 0.51 | 0.32-0.79 | ** | 0.87 | 0.51-1.51 |  | 0.73 | 0.43-1.22 |  | 0.72 | 0.39-1.33 |  |

ªReferent category.

Binary logistic regression analyses adjusted for clustering by school and maternal education level.

*P<0.05, **P<0.01, ***P<0.001
