# Supplementary material for: Family influences on children's physical activity and fruit and vegetable consumption
Source: Int J Behav Nutr Phys Act. 2009 Jun 16;6:34. doi: 10.1186/1479-5868-6-34 (PMC2703614; doi:10.1186/1479-5868-6-34)
Supplement: Additional file 3 — Table S3. Parental modelling and support items and the likelihood of combinations of physical activity and fruit and vegetable consumption among boys. The data provided present results from the multivariate multinomial logistic regression analyses examining the likelihood of combinations of physical activity and fruit and vegetable consumption among boys according to parental modelling and support items. [file 1479-5868-6-34-S3.doc]

**Table S3.** Parental modelling and support items and the likelihood of combinations of physical activity and fruit and vegetable consumption among boys.

|  | **Boys (n=354)** | | | | | |
| --- | --- | --- | --- | --- | --- | --- |
|  | **‘High PA / High FV’٭** | | **‘High PA / Low FV’٭** | | **‘Low PA / High FV’٭** | |
|  | **Unadjusted OR**  **(95% CI)§** | **Adjusted OR**  **(95% CI)†** | **Unadjusted OR**  **(95% CI)§** | **Adjusted OR**  **(95% CI)†** | **Unadjusted OR**  **(95% CI)§** | **Adjusted OR**  **(95% CI)†** |
| I/we did physical activity, sport or exercise together with the child |  |  |  |  |  |  |
| *Lowª* | 1.0 |  | 1.0 |  | 1.0 |  |
| *High* | 2.0 (1.1 to 3.6)* | 1.9 (1.0 to 3.4)* | 1.3 (0.7 to 2.5) |  | 1.2 (0.6 to 2.6) |  |
| I/ we ate breakfast at home together with the child |  |  |  |  |  |  |
| *Lowª* | 1.0 |  | 1.0 |  | 1.0 |  |
| *High* | 1.3 (0.7 to 2.3) |  | 0.7 (0.3 to 1.4) |  | 1.0 (0.5 to 2.4) |  |
| I/ we ate dinner at home together with the child |  |  |  |  |  |  |
| *Lowª* | 1.0 |  | 1.0 |  | 1.0 |  |
| *High* | 1.1 (0.5 to 2.1) |  | 0.7 (0.4 to 1.3) |  | 0.8 (0.4 to 1.5) |  |
| I/we took the child to sports training/lessons |  |  |  |  |  |  |
| *Lowª* | 1.0 |  | 1.0 |  | 1.0 |  |
| *High* | 1.3 (0.7 to 2.5) |  | 1.1 (0.6 to 1.8) |  | 1.0 (0.6 to 1.7) |  |
| I/we provided money for sport or physical activity that the child did |  |  |  |  |  |  |
| *Lowª* | 1.0 |  | 1.0 |  | 1.0 |  |
| *High* | 1.7 (0.8 to 3.6) |  | 1.1 (0.6 to 1.9) |  | 0.9 (0.5 to 1.7) |  |
| I/we provided money to the child to buy snacks, treats or fast food |  |  |  |  |  |  |
| *Lowª* | 1.0 |  | 1.0 |  | 1.0 |  |
| *Medium* | 1.4 (0.7 to 3.0) |  | 2.6 (1.4 to 5.1)** | 2.5 (1.3 to 5.0)** | 1.5 (0.6 to 4.0) |  |
| *High* | 1.1 (0.5 to 2.5) |  | 2.0 (1.1 to 3.7)* | 2.4 (0.9 to 6.6) | 0.9 (0.3 to 2.3) |  |
| I/we took the child to fast food restaurants |  |  |  |  |  |  |
| *Lowª* | 1.0 |  | 1.0 |  | 1.0 |  |
| *Medium* | 0.5 (0.3 to 1.2) | 0.5 (0.2 to 1.3) | 0.8 (0.4 to 1.7) |  | 1.0 (0.5 to 2.0) |  |
| *High* | 0.4 (0.2 to 1.0)* | 0.4 (0.1 to 1.3) | 0.6 (0.4 to 1.0) |  | 1.1 (0.6 to 2.0) |  |

ªReferent category. ٭Compared to ‘low PA/low FV’

§ Multinomial logistic regression analyses adjusted for clustering by school and maternal education only

† Adjusted for clustering by school, maternal education and all independent variables significantly related at the bivariate level

*P<0.05, **P<0.01
